# Grip strength, metabolic syndrome, and type 2 diabetes mellitus: a prospective study

**DOI:** 10.1007/s40200-025-01614-8

**Published:** 2025-05-10

**Authors:** JinWon Rho, DooYong Park, Yeon Soo Kim

**Affiliations:** 1 Department of Physical Education, College of Education, Seoul National University, Seoul, Republic of Korea; 2 Institute of Sports Science, Seoul National University, Seoul, Republic of Korea

**Keywords:** Grip strength, Muscle strength, Diabetes mellitus, Metabolic syndrome, Epidemiology

## Abstract

**Objectives:**

This study examines the impact of absolute grip strength (AGS) and relative grip strength (RGS) on the risk of developing type 2 diabetes mellitus (DM), especially in relation to metabolic syndrome (MetS).

**Methods:**

1,935 participants were adults aged 51 to 81 years with an average observation period of 6.50 years. The diagnosis of DM was based on American Diabetes Association. The JAMA 5030J1 dynamometer was used to measure the grip strength. Multivariable extended Cox regression models were used to calculate hazard ratios (HR) and 95% confidence intervals (CI) for DM incidence.

**Results:**

The DM risk increased with the number of MetS risk factors. High AGS was associated with a reduced DM risk (HR = 0.66, 95% CI = 0.44–0.99), but association disappeared when adjusted for MetS. High RGS was significantly associated with a reduced risk of DM incidence (HR = 0.49, 95% CI = 0.33–0.72), even after adjusting for MetS (HR = 0.63, 95% CI = 0.42–0.94). With the presence of MetS, higher AGS was associated with a greater increase in the DM risk compared to lower AGS, while higher RGS was associated with a less increase in the DM risk compared to lower RGS.

**Conclusion:**

This study demonstrates that RGS is a more reliable predictor of DM risks than AGS. Additionally, MetS significantly increases DM risk, particularly in individuals with obesity and hypertension. The study highlights the importance of assessing muscle quality in DM prevention and suggest that improving muscle quality may help mitigate DM risk.

## Introduction

The prevalence of type 2 diabetes mellitus (DM) has been emerging as a major public health issue [1]. DM is characterized by the inability to regulate blood sugar levels, which arises from insulin resistance [2]. The prevalence of DM among Korean adults has increased from 9.7% in 2012 to 13.6%. Among adults over 65 years, the prevalence is 30.7%, indicating that one in three individuals has DM.

Muscle strength is an important indicator of metabolic health, including DM [3]. Studies have shown that resistance exercise promotes health and prevents diseases [4]. Resistance exercise improves muscle strength, endurance and power, and helps manage DM, hypertension and obesity [5]. Absolute grip strength (AGS) is a commonly used measure to represent muscle strength and is widely used to evaluate overall health condition [6]. However, recent research suggests that relative grip strength (RGS), which is calculated by dividing AGS by body mass index (BMI, kg/m²), may be more effective measure for predicting risks of MetS and cardiovascular disease than AGS [7].

Previous study has shown that AGS and RGS are associated with the risk of developing DM [8]. However, most of the prior research has been cross-sectional, limiting the understanding of its long-term predictive value for DM risk. Additionally, while MetS is a well-established risk factor for DM, its potential role in modifying the relationship between AGS, RGS, and DM incidence has not been adequately explored. Given that muscle strength and metabolic health are interrelated, it is essential to determine whether RGS is a more reliable predictor of DM risk compared to AGS, particularly in individuals with MetS. Also, most of the researches were cross-sectional, suggesting the need for longitudinal research.

Therefore, this study aims to analyze the longitudinal impact of AGS and RGS on the risk of developing DM, especially examining differences according to the presence of MetS and the number of MetS risk factors. By clarifying these relationships, this study may provide valuable insights into the role of muscle function in DM prevention and help identify high-risk individuals who may benefit from targeted interventions.

## Methods

### Study population

The study analyzed the Korean Genome and Epidemiology Study (KoGES) data. The study targeted adults aged 51 to 81 years who participated in the baseline survey from 2013 to 2014 and the follow-up survey from 2019 to 2020. A total of 4,814 individuals were recruited. Individuals with DM or cardiovascular disease were excluded. Individuals with missing variables affecting DM, AGS and RGS were excluded as well. The final sample consisted of 1,935 participants with an average observation period of 6.50 years. The study was approved by the Institutional Review Boards of Korea University College of Medicine Ansan Hospital and Seoul National University, and written informed consent was obtained from the participants (IRB No. E2112/001–009).

### Measurements

#### DM

DM was defined if the fasting blood glucose level ≥ 126 mg/dL, the glycated hemoglobin level ≥ 6.5%, or affirmative responses to the questions “Have you been diagnosed with DM?” and “Are you currently taking oral DM medications?” [9, 10].

#### MetS risk factors and metabolic disorder

Obesity was defined if BMI ≥ 25 kg/m² according to previous study and Korean Society for the Study of Obesity’s guidelines [11, 12]. Hypertension was defined if systolic blood pressure (SBP) ≥ 140mmHG or diastolic blood pressure (DBP) ≥ 90mmHG or a diagnosis of hypertension or when currently taking antihypertensive medications for blood pressure management according to Korea hypertension fact sheet 2022 [13]. Hyperlipidemia was defined when triglyceride ≥ 150 mg/dL or total cholesterol ≥ 200 mg/dL or when currently taking lipid-lowering medications [14, 15].

#### AGS and RGS

The AGS test is simple to perform, provides prompt results, and is significantly associated with other measures of muscle strength. The JAMA 5030J1 dynamometer (SAEHAN, Korea) was used to measure the grip strength. Participants seated in a chair with their arms at a 90-degree angle while measuring grip strength. Each hand’s grip strength was measured three times, and the mean value was recorded. For the RGS, the AGS was divided by the BMI [9].

AGS and RGS were categorized into tertiles based on sex-specific distributions. Following previous research, AGS was classified into tertiles separately for males and females, with the lowest 0–33% classified as “Low,” the middle 34–66% as “Middle,” and the upper 67–100% as “High“ [16]. RGS was calculated as AGS (kg) divided by BMI (kg/m²) and also categorized into tertiles. The cutoff values for each category were determined separately for males and females to account for sex differences in grip strength.

#### Blood variables measurement

Participants maintained a fasting state for at least 8 h before serum collection. The blood samples collected were processed using a centrifuge on-site and sent to the Seoul Clinical Laboratory. The ADVIA 1800 auto-analyzer (Siemens, USA) was used to obtain DM related results from blood tests, including glucose, high sensitivity C-reactive protein (hs-CRP), serum creatinine, and insulin. The serum samples were uniformly processed and stored by the Genome Research Team of the Korea Centers for Disease Control and Prevention. The estimated glomerular filtration rate (eGFR), analyzed as a continuous variable, was calculated using the Modification of Diet in Renal Disease study formula based on serum creatinine level [17]. The homeostatic model assessment for Insulin resistance (HOMA-IR) value was calculated using the formula (fasting insulin × fasting blood glucose/405) [18], and insulin resistance levels were classified as “Low” if HOMA-IR ≥ 2.5 and “High” if < 2.5 [19, 20].

#### Questionnaire and other variables

Physical measurements included height, weight and BMI. The value of lean body mass was measured using the Zeus 9.9 device (JAWON Medical, Korea). The survey was conducted through one-on-one interviews by survey personnel and was reviewed and modified on the same day to enhance completeness. Alcohol consumption was assessed with the question, “Do you drink alcohol or have never consumed alcohol?”. Smoking status was assessed with, “Have you smoked more than 100 cigarettes in your lifetime?”. Physical activity participation was determined with, “Do you engage in regular exercise that makes you sweat?”. Household income level was assessed with, “What is the approximate monthly income of your household?”.

### Statistical analysis

STATA/IC 14.1(STATA corp., College Station, TX, USA) was used for data analysis. To examine the demographic characteristics of the study participants, frequency analysis was conducted using the chi-square test, and descriptive analysis was performed to calculate the mean values. Each variable was represented as a percentage or as mean and standard deviation. To ascertain the incidence density of DM among the observed participants, person-years were calculated over the entire follow-up period. To identify an appropriate analytical model for assessing the association between AGS, RGS, and the risk of DM incidence and the association between AGS, RGS, and the presence of MetS, a log-rank test was conducted for the proportional hazard assumption. The log-rank test results indicated significant findings for the proportional hazards assumption regarding MetS, RGS, and DM incidence (*p* < 0.05). The results for AGS were not significant (*p* = 0.93).

The current analytical model was deemed unsuitable as not all independent variables met the proportional hazard assumption. Therefore, to minimize biased results in data estimation by considering both time-fixed covariates and time-dependent covariates, the extended Cox regression model was used as the analysis model [21].

To examine the association between the number of MetS risk factors and the risk of DM incidence, the multivariable extended Cox regression model was used to calculate the independent hazard ratios (HR) and 95% confidence intervals (95% CI) of DM incidence according to the levels of AGS and RGS. The same model was used to compare the association between AGS, RGS, and the risk of DM incidence. Additionally, the interaction between the presence of MetS and AGS, RGS concerning the risk of DM incidence was compared. In the multivariable extended Cox regression model analysis, confounding variables were adjusted, including age, sex, exercise participation, skeletal muscle mass, eGFR, hs-CRP, alcohol consumption, smoking status, income level, and HOMA-IR. All significance levels were set at *p* < 0.05.

## Results

The demographic characteristics are shown in Table 1. Significant differences were observed in all variables except for sex between the low AGS group and the high AGS group. Significant differences were also observed in all variables except for sex and the prevalence of hyperlipidemia between the low RGS group and the high RGS group.

**Table 1:** Baseline characteristics of study participants

Characteristic of risk factor	AGS			RGS		
	Low AGS (*n* = 920)	High AGS (*n* = 1,015)	*p*	Low RGS (*n* = 953)	High RGS (*n* = 982)	*p*
Age (years)	69.32 ± 7.70	61.43 ± 6.71	< 0.001	68.31 ± 7.84	62.15 ± 7.38	< 0.001
Male (%)	44.89	41.38	0.119	43.13	42.97	0.946
Lean body mass (kg)	38.23 ± 7.14	42.22 ± 8.05	< 0.001	39.43 ± 7.64	41.18 ± 8.03	< 0.001
Income status (< 1 million won,)	62.83	33.79	< 0.001	60.02	35.54	< 0.001
eGFR (mL/min per 1.73 m²)	94.11 ± 22.18	96.57 ± 20.47	0.011	93.93 ± 22.15	96.83 ± 20.42	0.003
hs-CRP (mg/dL)	1.71 ± 4.06	1.33 ± 3.57	0.030	1.74 ± 3.98	1.29 ± 3.64	0.009
Alcohol Consumption (%)						
None	54.46	49.95	< 0.001	54.04	50.20	< 0.001
Former	9.89	5.52		9.44	5.80	
Current	35.65	44.53		36.52	43.99	
Smoking Status (%)						
None	64.78	68.87	0.014	66.32	67.52	< 0.001
Former	23.48	18.13		24.13	17.31	
Current	11.74	13.00		9.55	15.17	
Exercise Participation (%)						
None	76.63	63.84	< 0.001	75.13	64.87	< 0.001
Regular	23.37	36.16		24.87	35.13	
HOMA-IR	1.87 ± 0.87	1.97 ± 1.06	0.020	2.07 ± 1.14	1.79 ± 0.75	< 0.001
Metabolic Syndrome (%)	23.59	29.78	0.002	32.21	21.61	< 0.001
Obesity (%)	32.61	43.84	< 0.001	50.37	26.99	< 0.001
Hypertension (%)	55.76	49.06	0.003	60.44	44.30	< 0.001
Dyslipidemia (%)	49.57	54.68	0.024	52.57	51.93	0.779

Abbreviation: AGS, absolute grip strength; BMI, body mass index; eGFR, estimated glomerular filtration rate; HOMA-IR, homeostatic model assessment for Insulin resistance; HR, hazard ratio; hs-CRP, high sensitivity-C-reactive protein; RGS, relative grip strength

The association between the number of MetS risk factors and the presence of MetS with DM incidence are shown in Table 2. The incidence density of DM per 1,000 people increased with the number of MetS risk factors. When various confounding variables and AGS were adjusted, the HR for DM incidence also significantly increased (p-trend < 0.001). When all five MetS risk factors were present, the DM incidence HR was 20.74 times higher than no MetS risk factors were present (HR = 20.74, 95% CI = 7.91–54.38). Additionally, incidence densities of DM per 1,000 people for groups with MetS, obesity, and hypertension were 3.21, 1.97, and 1.63 times higher, respectively, compared to those without conditions. When confounding variables and AGS were adjusted, the DM incidence HR was 2.98 (HR = 2.98, 95% CI = 2.22-4.00), 1.71 (HR = 1.71, 95% CI = 1.25–2.33), and 1.71 times (HR = 1.71, 95% CI = 1.27–2.31) higher, respectively, compared to those without conditions. However, there were no significant results regarding the risk of DM incidence and hyperlipidemia. Additionally, similar results were observed in Model 1.

**Table 2:** Incidence density and hazard ratio of DM according to number of MetS risk factor and metabolic disorder

Characteristic of risk factors		DM (*n* = 1,935)	Person-year	Incidence density* (95% CI)	Multivariable for HR (95% CI)	Model 1
Number of MetS Risk factor	0	9	1742.60	5.16 (2.68, 9.92)	1.00 (Reference)	1.00 (Reference)
	1	31	2473.11	12.53 (881, 17.82)	**2.54** (1.20, 5.35)	**2.40** (1.14, 5.06)
	2	55	3138.46	17.52 (13.45, 22.82)	**3.63** (1.77, 7.42)	**3.32** (1.62, 6.80)
	3	52	1758.97	29.56 (22.52, 38.29)	**5.55** (2.69, 11.48)	**5.58** (2.69, 11.57)
	4	47	699.12	67.22 (50.51, 89.47)	**13.07** (6.28, 27.22)	**12.06** (5.75, 25.29)
	5	9	89.80	100.21 (52.14, 192.60)	**20.74** (7.91, 54.38)	**20.39** (7.81, 53.24)
p- trend					< 0.001	< 0.001
MetS	No	95	7354.18	12.91 (10.56, 15.79)	1.00 (Reference)	1.00 (Reference)
	Yes	108	2547.90	42.38 (35.10, 51.18)	**2.98** (2.22, 4.00)	**3.03** (2.26, 4.07)
p- trend					< 0.001	< 0.001
Obesity	No	91	6109.55	14.89 (12.12, 18.29)	1.00 (Reference)	1.00 (Reference)
	Yes	112	3798.45	29.48 (24.50, 35.48)	**1.71** (1.25, 2.33)	**1.59** (1.14, 2.21)
p- trend					0.001	0.005
Hypertension	No	75	4839.18	15.49 (12.35, 19.43)	1.00 (Reference)	1.00 (Reference)
	Yes	128	5068.82	25.25 (21.23, 30.02)	**1.71** (1.27, 2.31)	**1.67** (1.24, 2.25)
p- trend					< 0.001	0.001
Dyslipidemia	No	83	4742.27	17.50 (14.11, 21.70)	1.00 (Reference)	1.00 (Reference)
	Yes	120	5165.73	23.22 (19.42, 27.78)	1.20 (0.90, 1.60)	1.20 (0.91, 1.60)
p- trend					0.201	0.189
Total		203	9908.00	20.48		

*Incidence density = case/person-year×1,000

Multivariable model adjusted age, sex, eGFR, hs-CRP, lean body mass, alcohol intake, smoking status, income status, HOMA-IR, exercise participation, AGS

Model1, plus adjusted by changing AGS to RGS

Abbreviation: AGS, absolute grip strength; BMI, body mass index; DM, type 2 diabetes mellitus; eGFR, estimated glomerular filtration rate; HOMA-IR, homeostatic model assessment for Insulin resistance; HR, hazard ratio; hs-CRP, high sensitivity-C-reactive protein; RGS, relative grip strength

The association between the levels of AGS, RGS, and DM incidence are presented in Table 3. The high AGS group showed a lower incidence density per 1,000 people compared to the low AGS group, and the HR for DM incidence reduced by 34% (HR = 0.66, 95% CI = 0.44–0.99). However, in Model 1, adjusted for the presence of MetS, the association between AGS and the risk of DM incidence disappeared. The high RGS group showed a lower incidence density per 1,000 people compared to the low RGS group, and the HR for DM incidence reduced by 51% (HR = 0.49, 95% CI = 0.33–0.72). Additionally, in Model 1, a significant association between high RGS and the risk of DM incidence was observed (HR = 0.63, 95% CI = 0.42–0.94).

**Table 3:** Association of AGS and RGS with risk of developing DM

Characteristic of risk factors		DM (*n* = 1,935)	Person-year	Incidence density* (95% CI)	Multivariable for HR (95% CI)	Model1
AGS (kg)	Low	62	2954.76	20.98 (16.35, 26.91)	1.00 (Reference)	1.00 (Reference)
	Middle	65	3305.08	19.66 (15.42, 25.07)	**0.81** (0.56, 0.17)	0.78 (0.54, 1.13)
	High	76	3648.15	20.83 (16.63, 26.08)	**0.66** (0.44, 0.99)	0.68 (0.45, 1.03)
p- trend					0.047	0.078
RGS (kg/BMI)	Low	71	3069.97	23.12 (18.32, 29.18)	1.00 (Reference)	1.00 (Reference)
	Middle	79	3296.16	23.96 (19.22, 29.88)	0.90 (0.64, 1.25)	0.99 (0.71, 1.39)
	High	53	3541.86	14.96 (11.43, 19.58)	**0.49** (0.33, 0.72)	**0.63** (0.42, 0.94)
p- trend					< 0.001	0.027
Total		203	9908.00	20.48		

*Incidence density = case/person-year×1,000

Multivariable model adjusted age, sex, eGFR, hs-CRP, lean body mass, alcohol intake, smoking status, income status, HOMA-IR, exercise participation

Model1, plus adjusted MetS

Abbreviation: AGS, absolute grip strength; BMI, body mass index; DM, type 2 diabetes mellitus; eGFR, estimated glomerular filtration rate; HOMA-IR, homeostatic model assessment for Insulin resistance; HR, hazard ratio; hs-CRP, high sensitivity-C-reactive protein; RGS, relative grip strength

**Fig. 1:**
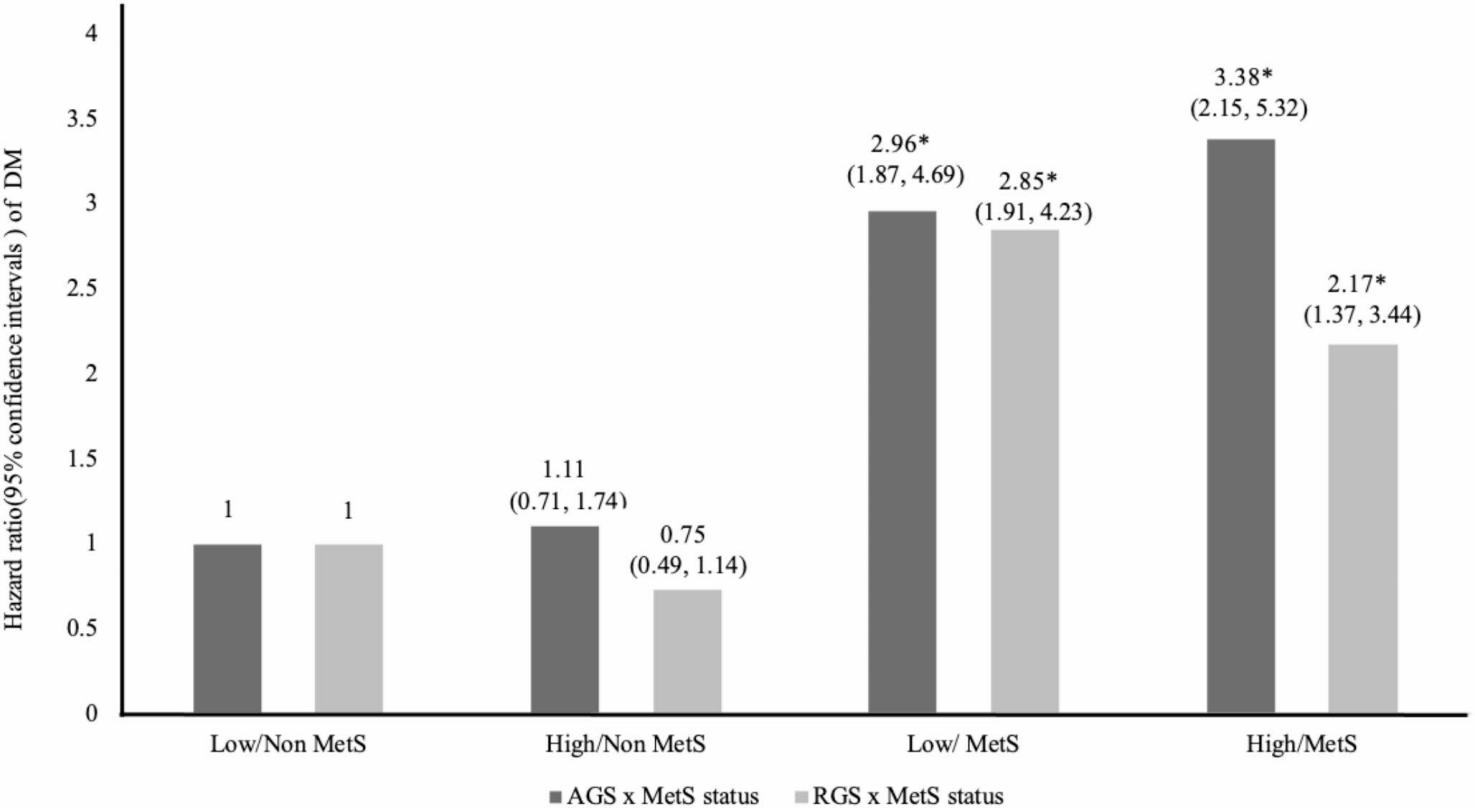
Joint association of AGS, RGS and MetS status with risk of developing DM. Multivariable model adjusted age, sex, eGFR, hs-CRP, lean body mass, alcohol intake, smoking status, income status, HOMA-IR, exercise participation, Abbreviation: AGS, absolute grip strength; DM, diabetes mellitus; eGFR, estimated glomerular filtration rate; HOMA-IR, homeostatic model assessment for insulin resistance; hs-CRP, high sensitivity-C-reactive protein; MetS, metabolic syndrome; RGS, relative grip strength *P < 0.05, **p < 0.01, ***p < 0.001

The association between the risk of DM incidence and the association of MetS status with AGS and RGS are presented in Fig. 1. Compared to the non-MetS group with low AGS and, the risk of DM incidence was 2.96 times higher (HR = 2.96, 95% CI = 1.87–4.69) for the MetS group with low AGS and 3.38 times higher (HR = 3.38, 95% CI = 2.15–5.32) for the MetS group with high AGS. Additionally, compared to the non-MetS group with low RGS, the risk of DM incidence was 2.85 times higher (HR = 2.85, 95% CI = 1.91–4.23) for the MetS group with low RGS, and 2.17 times higher (HR = 2.17, 95% CI = 1.37–3.44) for the MetS group with high RGS. With the presence of MetS, a higher AGS is associated with a greater increase in the risk of DM compared to a lower AGS, while a higher RGS is associated with a smaller increase in the risk of DM compared to a lower RGS.

## Discussion

This study confirmed association between MetS risk factors and metabolic disorders with the DM incidence. Specifically, the risk of DM incidence increased with the number of MetS risk factors and the presence of MetS. Additionally, both high AGS and RGS groups showed a reduced risk of DM incidence. However, when adjusted for the presence of MetS the association between AGS and the risk of DM incidence disappeared, while RGS remained a significant predictor. Furthermore, a significant association was observed between AGS, RGS, and MetS status concerning the risk of DM incidence.

Previous research has established a positive association between MetS and the risk of developing DM. A longitudinal study conducted on the Japanese population found that MetS components significantly predict the incidence of type 2 DM [22]. The risk of DM increased progressively with a higher number of MetS risk factors. The multivariable-adjusted HRs for developing DM were 2.00 (95% CI = 1.60–2.60) for one risk factor, 4.30 (95% CI = 3.40–5.40) for two risk factors, 7.0 (95% CI = 5.30–9.20) for three risk factors, and 10.00 (95% CI = 6.40–15.80) for four risk factors, compared to non-MetS [22]. Similarly, findings from the European Prospective Investigation into Cancer and Nutrition-Potsdam Study highlighted that MetS markedly increases the risk of DM, underscoring the importance of monitoring MetS components to predict the prevalence of DM [23]. MetS was a significant predictor of DM onset, with an adjusted HR of 4.62 (95% CI = 3.90–5.48). In a sub-analysis of participants with fasting blood samples (*N* = 788, 189 events), the adjusted HR increased to 4.83 (95% CI = 3.46–6.75) [23].

Previous longitudinal studies have shown that MetS is a key predictor of DM incidence, with a progressive increase in risk as the number of MetS risk factors increases. These findings highlight the importance of monitoring MetS to mitigate DM risk. This study aligns with these findings, demonstrating that while AGS initially appears protective against DM, its predictive power diminishes when adjusted for metabolic factors. This suggests that AGS may be influenced by body mass and overall metabolic status rather than serving as an independent marker of diabetes risk. In contrast, RGS remains as an independent predictor of lower DM incidence, reinforcing its potential as a more reliable muscle quality marker.

Grip strength is widely recognized as an accessible and reliable indicator of overall neuromuscular health, particularly in adults [24]. Additionally, muscle strength is linked to the maintenance of skeletal muscle mass, which helps reduce the risk of various diseases [25]. Studies have shown that muscle strength is strongly correlated with mobility, functional status, and mortality in the elderly, regardless of muscle mass and RGS is known as a method that can predict sarcopenia due to decreased functional ability of muscles [26]. Also, previous study has shown that RGS is associated with the risk of DM incidence and is a better predictor than AGS [8]. Another study highlighted the importance of RGS as a predictor for MetS, showing that higher RGS is consistently associated with lower risk of MetS [27].

The findings from Table 2 indicate that MetS independently increase the risk of developing DM incidence, even after adjusting for confounding variables, including AGS and RGS. This can be explained by the greater impact of MetS on risk of developing DM compared to the impact of grip strength. Notably, obesity and hypertension have shown a strong association with DM, whereas hyperlipidemia appears to have less association, which shows similar results from the previous studies [28–31].

The results from Table 3 indicated that association between the high AGS group and DM incidence disappeared when adjusted for MetS, whereas the high RGS group maintained its significance. This suggests that AGS is mediated by MetS risk factors in its association with DM incidence [32]. In prior research comparing AGS and RGS in relation to the incidence of DM, it was found that the low AGS independently raised the risk of DM by 1.21 times in women and 1.34 times in men, while the lowest RGS elevated the risk by 6.56 times in women and 5.31 times in men [33]. Additionally, another study revealed that a 0.1 unit increase in RGS decreased the risk of DM by 19% [34]. Also, AGS is closely related to Mets risk factors, which can influence the incidence of DM [35].

As shown in Table 1, the high AGS group had higher proportions of MetS, obesity, and dyslipidemia compared to the low AGS group. Conversely, the high RGS group had lower proportions of MetS, obesity, and hypertension compared to the low RGS group, which is consistent with previous research [35]. The study suggests that association of AGS with DM risk is mediated by MetS risk factors, while RGS remains a robust predictor of DM prevalence risk, independent of MetS. These results highlight the importance of considering muscle quality, as represented by RGS, in evaluating DM risk, and suggest that interventions aimed at improving muscle quality could be beneficial in reducing DM risk [35].

In Fig. 1, with the presence of MetS, a higher AGS is associated with a greater increase in the risk of DM incidence compared to a lower AGS, while a higher RGS is associated with a smaller increase in the risk of DM incidence compared to a lower RGS. The risk of DM incidence increases in the presence of MetS despite having a high RGS, but to a less extent compared to lower RGS, which could be attributed to the quality of muscle. High RGS indicates better muscle quality, which is associated with lower insulin resistance [3]. Improved muscle quality can help mitigate the adverse effects of MetS on DM risk, reducing insulin resistance and improving overall metabolic health. This underscores the protective role of high RGS against the development of DM. AGS tends to increase with body weight, making high AGS a potential indicator of obesity rather than muscle health, complicating its use in predicting DM risk [36]. Consequently, RGS tends to be a better predictor of DM risk as it more accurately reflects muscle quality and health [8]. Therefore, high RGS can mitigate the direct adverse effects of MetS on the increased risk of developing DM.

This study is a significant longitudinal study showing the impact of AGS and RGS on the risk of developing DM, especially examining the differences according to the presence of MetS and number of the risk factors. Also, the findings of this study have significant clinical and public health implications. While resistance training interventions often emphasize increasing muscle mass, our results suggest that improving RGS may be more beneficial for metabolic health. Future research should explore the mechanisms linking RGS to insulin sensitivity and glucose metabolism, particularly in individuals with metabolic syndrome.

However, this study has several limitations. First, dietary factors that affect DM or MetS were not adjusted for in this analysis. However, lifestyle habits such as smoking, alcohol consumption, exercise, and blood variables were adjusted to enhance the reliability of this study. Also, there is a limitation in generalizing the findings of this study, as the study participants were adults from specific regions of South Korea. Future studies should investigate a nationwide cohort study. Third, this study only utilized grip strength. Thus, it was unable to ascertain the associations between muscle strength in other regions, such as the lower body and abdomen, and risk of developing DM. Future research should aim to separately investigate the effects of AGS and RGS in the lower body, and abdomen muscles as well.

Additionally, the duration of DM might have had an impact. Long-term DM patients often experience progressive muscle loss and insulin resistance, which might have an impact on grip strength measures differently compared to recently diagnosed individuals. Future studies should consider stratifying participants based on duration of DM incidence to further elucidate its impact on muscle strength and MetS risk.

## Conclusion

This study highlights the significant associations between MetS risk factors, AGS and RGS, and the risk of developing DM. The findings underscore that both high AGS and RGS are associated with a reduced risk of DM incidence. However, the effect of AGS on DM risk disappears when adjusted for MetS, whereas RGS maintains its significance. This suggests that RGS is a more reliable predictor of DM risk due to its reflection of muscle quality rather than sheer muscle mass.

The study also emphasizes that MetS significantly increases the risk of developing DM. The progressive increase in DM risk with the number of MetS components aligns with previous research, reaffirming the necessity to monitor and manage MetS components to mitigate DM risk. Specifically, conditions like obesity and hypertension show a stronger association with DM, compared to hyperlipidemia. These results suggest that interventions aimed at improving muscle quality could be effective in reducing the risk of DM, particularly in individuals with MetS.

## Data Availability

Data will be made available on reasonable request.

## References

[1] Tomic D, Shaw JE, Magliano DJ. The burden and risks of emerging complications of diabetes mellitus. Nat Reviews Endocrinol. 2022;18(9):525–39. 10.1038/s41574-022-00690-7 PMC9169030 35668219

[2] Siddiqui AA, Siddiqui SA, Ahmad S, Siddiqui S, Ahsan I, Sahu K. Diabetes: mechanism, pathophysiology and management-A review. Int J Drug Dev Res. 2013;5(2):1–23.

[3] Lee M-R, Jung SM, Bang H, Kim HS, Kim YB. Association between muscle strength and type 2 diabetes mellitus in adults in Korea: data from the Korea National health and nutrition examination survey (KNHANES) VI. Medicine. 2018;97(23):e10984. 10.1097/MD.0000000000010984. 29879054 10.1097/MD.0000000000010984 PMC5999476

[4] D’Onofrio G, Kirschner J, Prather H, Goldman D, Rozanski A. Musculoskeletal exercise: its role in promoting health and longevity. Prog Cardiovasc Dis. 2023;77:25–36. 10.1016/j.pcad.2023.02.006. 36841491 10.1016/j.pcad.2023.02.006

[5] Tresierras MA, Balady GJ. Resistance training in the treatment of diabetes and obesity: mechanisms and outcomes. J Cardiopulm Rehabil Prev. 2009;29(2):67–75. 19305230 10.1097/HCR.0b013e318199ff69

[6] Sasaki H, Kasagi F, Yamada M, Fujita S. Grip strength predicts cause-specific mortality in middle-aged and elderly persons. Am J Med. 2007;120(4):337–42. 17398228 10.1016/j.amjmed.2006.04.018

[7] Churilla JR, Summerlin M, Richardson MR, Boltz AJ. Mean combined relative grip strength and metabolic syndrome: 2011–2014 National health and nutrition examination survey. J Strength Conditioning Res. 2020;34(4):995–1000. 10.1519/JSC.0000000000003515. 10.1519/JSC.0000000000003515 31996611

[8] Park D, Rho J, Kim E, Kim Y. Comparison of absolute and relative grip strength to predict incidence of diabetes mellitus in Korea: A prospective cohort study. Metabolic Syndrome and Related Disorders; 2024. 10.1089/met.2024.0006 38634825

[9] Kim GH, Song BK, Kim JW, Lefferts EC, Brellenthin AG, Lee D-c, et al. Associations between relative grip strength and type 2 diabetes mellitus: the Yangpyeong cohort of the Korean genome and epidemiology study. PLoS ONE. 2021;16(8):e0256550. 10.1371/journal.pone.0256550. 34437604 10.1371/journal.pone.0256550 PMC8389482

[10] Wu H, Gu Y, Wang X, Meng G, Rayamajhi S, Thapa A, et al. Association between handgrip strength and type 2 diabetes: A prospective cohort study and systematic review with meta-analysis. Journals Gerontology: Ser A. 2023;78(8):1383–91. 10.1093/gerona/glac241 36504134

[11] Nam GE, Kim Y-H, Han K, Jung J-H, Rhee E-J, Lee W-Y. Obesity fact sheet in Korea, 2020: prevalence of obesity by obesity class from 2009 to 2018. J Obes Metabolic Syndrome. 2021;30(2):141. 10.7570/jomes21056 PMC8277583 34158420

[12] Seo MH, Lee W-Y, Kim SS, Kang J-H, Kang J-H, Kim KK, et al. 2018 Korean society for the study of obesity guideline for the management of obesity in Korea. J Obes Metabolic Syndrome. 2019;28(1):40. 10.7570/jomes.2019.28.1.40 PMC6484940 31089578

[13] Kim H-L, Lee EM, Ahn SY, Kim K-i, Kim HC, Kim JH, et al. The 2022 focused update of the 2018 Korean hypertension society guidelines for the management of hypertension. Clin Hypertens. 2023;29(1):11. 10.1186/s40885-023-00234-9. 36788612 10.1186/s40885-023-00234-9 PMC9930285

[14] Krüger K, Tirekoglou P, Weyh C. Immunological mechanisms of exercise therapy in dyslipidemia. Front Physiol. 2022;13:903713. 10.3389/fphys.2022.903713. 36003652 10.3389/fphys.2022.903713 PMC9393246

[15] Detection, NCEPEPo. Third report of the National cholesterol education program (NCEP) expert panel on detection, evaluation, and treatment of high blood cholesterol in adults (Adult treatment panel III). Adults ToHBCi. The Program; 2002. 12485966

[16] Lopez-Lopez JP, Cohen DD, Ney-Salazar D, Martinez D, Otero J, Gomez-Arbelaez D, et al. The prediction of metabolic syndrome alterations is improved by combining waist circumference and handgrip strength measurements compared to either alone. Cardiovasc Diabetol. 2021;20:1–11. 33752666 10.1186/s12933-021-01256-z PMC7986558

[17] Andrassy KM. Comments on ‘KDIGO 2012 clinical practice guideline for the evaluation and management of chronic kidney disease’. Kidney Int. 2013;84(3):622–3. 10.1038/ki.2013.243. 23989362 10.1038/ki.2013.243

[18] Parker K, Tucker LA, editors. The role of physical activity in the relationship between sitting time and insulin resistance. International Journal of Exercise Science: Conference Proceedings; 2021.

[19] George AK, Narayan V, Kurian N, Joseph AE, Anil S. A pilot study on glycemia and insulin resistance in patients with severe periodontitis. J Indian Soc Periodontology. 2021;25(5):393–8. 10.4103/jisp.jisp_419_20. 10.4103/jisp.jisp_419_20 PMC8452164 34667381

[20] Lim SG, Han K, Kim HA, Pyo SW, Cho YS, Kim KS, et al. Association between insulin resistance and periodontitis in Korean adults. J Clin Periodontol. 2014;41(2):121–30. 10.1111/jcpe.12196. 24303984 10.1111/jcpe.12196

[21] Baik SH, Fung K-W, McDonald CJ. The mortality risk of proton pump inhibitors in 1.9 million US seniors: an extended Cox survival analysis. Clin Gastroenterol Hepatol. 2022;20(4):e671–81. 10.1016/j.cgh.2021.01.014. 33453399 10.1016/j.cgh.2021.01.014 PMC12381940

[22] Kurotani K, Miyamoto T, Kochi T, Eguchi M, Imai T, Nishihara A, et al. Metabolic syndrome components and diabetes incidence according to the presence or absence of impaired fasting glucose: the Japan epidemiology collaboration on occupational health study. J Epidemiol. 2017;27(9):408–12. 10.1016/j.je.2016.08.015. 28434837 10.1016/j.je.2016.08.015 PMC5565752

[23] Ford ES, Schulze MB, Pischon T, Bergmann MM, Joost H-G, Boeing H. Metabolic syndrome and risk of incident diabetes: findings from the European prospective investigation into cancer and Nutrition-Potsdam study. Cardiovasc Diabetol. 2008;7:1–8. 10.1186/1475-2840-7-35. 19077281 10.1186/1475-2840-7-35 PMC2627822

[24] Visser M, Deeg DJ, Lips P, Harris TB, Bouter LM. Skeletal muscle mass and muscle strength in relation to lower-extremity performance in older men and women. J Am Geriatr Soc. 2000;48(4):381–6. 10798463 10.1111/j.1532-5415.2000.tb04694.x

[25] Choquette S, Bouchard D, Doyon C, Sénéchal M, Brochu M, Dionne IJ. Relative strength as A determinant of mobility in elders 67–84 years of age. A Nuage study: nutrition as A determinant of successful aging. J Nutr Health Aging. 2010;14:190–5. 10.1007/s12603-010-0047-4.20191251 10.1007/s12603-010-0047-4

[26] Fragala MS, Kenny AM, Kuchel GA. Muscle quality in aging: a multi-dimensional approach to muscle functioning with applications for treatment. Sports Med. 2015;45:641–58. 10.1007/s40279-015-0305-z. 25655372 10.1007/s40279-015-0305-z

[27] Park D, Rho J, Kim Y, Kim E. Comparison of absolute and relative grip strength to predict incidence of metabolic syndrome: Eight-Year Follow-Up study in Korea. Metabolic Syndrome and Related Disorders; 2024. 10.1089/met.2023.0206 38227796

[28] Banegas JR, López-García E, Graciani A, Guallar-Castillón P, Gutierrez-Fisac JL, Alonso J, et al. Relationship between obesity, hypertension and diabetes, and health-related quality of life among the elderly. Eur J Prev Cardiol. 2007;14(3):456–62. 10.1097/HJR.0b013e3280803f29. 10.1097/HJR.0b013e3280803f29 17568249

[29] Seidell JC. Obesity, insulin resistance and diabetes—a worldwide epidemic. Br J Nutr. 2000;83(S1):S5–8. 10889785 10.1017/s000711450000088x

[30] Vekic J, Zeljkovic A, Stefanovic A, Jelic-Ivanovic Z, Spasojevic-Kalimanovska V. Obesity and dyslipidemia. Metabolism. 2019;92:71–81. 30447223 10.1016/j.metabol.2018.11.005

[31] Saleem Z, Saeed H, Khan ZA, Khan MIH, Hashmi FK, Islam M, et al. Association of hypertension and dyslipidaemia with increasing obesity in patients with type 2 diabetes mellitus. Brazilian J Pharm Sci. 2019;55:e18136.

[32] Merchant RA, Chan YH, Lim JY, Morley JE. Prevalence of metabolic syndrome and association with grip strength in older adults: findings from the HOPE study. Diabetes, metabolic syndrome and obesity. 2020:2677–86. doi: 10.2147/DMSO.S260544 10.2147/DMSO.S260544 PMC7419634 32821140

[33] Boonpor J, Parra-Soto S, Petermann-Rocha F, Ferrari G, Welsh P, Pell J, et al. Associations between grip strength and incident type 2 diabetes: findings from the UK biobank prospective cohort study. BMJ Open Diabetes Res Care. 2021;9(1):e001865. Epub 2021/08/07. PMID: 34353878; 2021. 34353878 10.1136/bmjdrc-2020-001865 PMC8344322

[34] Karvonen-Gutierrez CA, Peng Q, Peterson M, Duchowny K, Nan B, Harlow S. Low grip strength predicts incident diabetes among mid-life women: the Michigan study of women’s health across the Nation. Age Ageing. 2018;47(5):685–91. 10.1093/ageing/afy067. 29726885 10.1093/ageing/afy067 PMC6108393

[35] Byeon JY, Lee MK, Yu M-S, Kang MJ, Lee DH, Kim KC, et al. Lower relative handgrip strength is significantly associated with a higher prevalence of the metabolic syndrome in adults. Metab Syndr Relat Disord. 2019;17(5):280–8. 10.1089/met.2018.0111. 30945974 10.1089/met.2018.0111

[36] Fahs CA, Thiebaud RS, Rossow LM, Loenneke JP, Bemben DA, Bemben MG. Relationships between central arterial stiffness, lean body mass, and absolute and relative strength in young and older men and women. Clin Physiol Funct Imaging. 2018;38(4):676–80. 10.1111/cpf.124676ss76. 28815984 10.1111/cpf.12467

